# Complementary Feeding and Growth in Infants Born Preterm: A 12 Months Follow-Up Study

**DOI:** 10.3390/children8121085

**Published:** 2021-11-24

**Authors:** Giovanni Boscarino, Maria Giulia Conti, Federica Pagano, Maria Di Chiara, Chiara Pannucci, Elisa Onestà, Rita Prota, Giorgia Deli, Lucia Dito, Daniela Regoli, Salvatore Oliva, Gianluca Terrin

**Affiliations:** 1 Department of Maternal and Child Health, Policlinico Umberto I, Sapienza University of Rome, 00161 Rome, Italy; giovanni.boscarino@yahoo.com (G.B.); mariagiulia.conti@uniroma1.it (M.G.C.); federica.pagano22@gmail.com (F.P.); maria.dichiara@uniroma1.it (M.D.C.); chiara.pannucci@gmail.com (C.P.); elisa.onesta@gmail.com (E.O.); rita-prota@libero.it (R.P.); giorgia.deli7@gmail.com (G.D.); lucia.dito@yahoo.it (L.D.); dani.regoli@virgilio.it (D.R.); salvatore.oliva@uniroma1.it (S.O.); 2 Department of Molecular Medicine, Sapienza University of Rome, 00185 Rome, Italy

**Keywords:** weaning, nutrition, VLBW, body weight, length, body mass index, follow-up, complementary food, breastfeeding, infant formula, neonatology, microflora

## Abstract

Evidences demonstrated that timing of weaning influences long-term growth in full term infants. However, studies on preterm infants are still lacking, and the international guidelines are focused only on healthy full-term newborn, without consensus for preterms. We aimed at evaluating, in a cohort study, the consequences of different timing of weaning on auxological outcomes up to 12 months of corrected age in a population of neonates born with gestational age < 32 weeks or birth weight < 1500 g. We divided the enrolled neonates in two cohorts according to the timing of weaning: (i) Early Weaning: introduction of complementary food before 6 months of corrected age; (ii) Late Weaning: complementary food introduced after 6 months of corrected age. Growth parameters (weight, length, body mass index, and ponderal index) were measured at 12 months of life. The two groups were statistically comparable for baseline clinical characteristics, and differences on growth parameters were not reported between the two study groups. These results were confirmed in linear and binary logistic regression multivariate models. Timing of weaning is not related to growth of preterm newborns in the first 12 months of corrected age. Studies are needed to reach consensus for the appropriate nutritional approach for preterm babies after discharge.

## 1. Introduction

According to the World Health Organization (WHO), survival of preterm infants has significantly improved thanks to the advances in neonatal care [1]. With the improvement in neonatal survival, preterm birth rates are increasing.

One of the most important prematurity-related morbidity in survived infants is malnutrition, which is in turn associated with poorer growth [2]. It has been demonstrated that there are critical windows for nutritional intervention during the first months of life, which may influence long-term growth. [2,3,4,5,6,7]. Evidences for healthy, full term infants demonstrated that the timing of starting complementary feeding, known as weaning, influences growth outcomes [8]. The WHO defines weaning the period when the diet changes from complete breastfeeding to when the child is able to eat family food [9]. International nutritional guidelines recommend exclusive or predominant breastfeeding approximately for the first six months [10,11]. However, timing and modalities for the introduction of complementary food are still lacking in preterm infants [12]. International guidelines are focused mainly on healthy full-term newborn, without consensus for preterms and, consequently, with variable clinical attitude among physicians [10,11].

Studies on preterm infants recognized that the first weeks of life represent a crucial timeframe for nutritional intervention [2,3,5,13], while it is yet to be demonstrated if the timing of weaning may also be considered an additional critical window for long-term growth outcomes [10]. Studies on optimal timing of weaning in preterms are needed; we designed a cohort study to evaluate the consequences of weaning on auxological outcomes of infants, born very preterm, in the first 12 months of corrected age.

## 2. Materials and Methods

We enrolled all newborns with gestational age < 32 weeks or body birth weight < 1500 g, consecutively admitted in Neonatal Intensive Care Unit (NICU) of Policlinico Umberto I, Sapienza University of Rome, from January 2015 to December 2019 and with a follow-up of 12 months. We excluded subjects with major congenital (intestinal and extra-intestinal) malformations, inborn errors of metabolism, born to mother with autoimmune diseases, congenital infection, transfer to other hospital or death within the first 72 h of life, or with incomplete clinical data, lost to follow-up, or that developed food intolerance in the first 12 months [14,15,16,17,18,19,20].

Researchers not involved in the clinical practice collected data in a specific data form, unaware of the study aims; a statistician blinded to the study aims performed data analysis. We prospectively recorded prenatal, perinatal, and postnatal data in a specific data form. All infants were monitored until discharge, transfer to other hospital, or death. Maternal age, gestational age, and body weight at birth, antenatal steroid administration, type of delivery, gender, twin pregnancies, 5-min Apgar score, pH on cord blood, and Clinical Risk Index for Babies (CRIB) II score were collected [21]. Morbidity was defined as the presence of at least one of the major prematurity-related complications, such as necrotizing enterocolitis (NEC) Bell-Stage ≥ 2, intraventricular hemorrhage (IVH), periventricular leukomalacia (PVL), sepsis proven by positive culture, retinopathy of prematurity (ROP), and bronchopulmonary dysplasia (BPD) of at least moderate grade. Diagnosis of NEC, BPD, IVH, PVL, ROP, and sepsis were performed according to standard criteria by physicians caring for the babies and were blinded of the study aims [22,23,24,25,26]. Extrauterine growth restriction (EUGR) with longitudinal definition was defined as the loss of 1 standard deviation from birth to 36 weeks of PMA [27]. The age of 36 weeks of PMA represents a criteria for discharge. Data on PN and EN were daily collected. We also collected data regarding need of invasive or non-invasive mechanical ventilation.

At 52 weeks of postmenstrual age (PMA) and at 12 months of corrected age, nurses unaware of the study aims measured growth parameters (body weight, length, body mass index (BMI, weight/length^2^), and ponderal index (weight/length^3^)), as previously described [28]. Enteral and parenteral nutrition from hospital admission to discharge were administered as previously described [29,30]. At discharge, medical staff encouraged breastfeeding. Preterm formula was administered when breast milk was insufficient or not available up to 2500 g of body weight. Subsequently, at term, formula was introduced.

At discharge, parents and family pediatricians of babies were provided with a detailed set of written recommendations regarding modalities of introduction of complementary food, in accordance with European Society for Pediatric Gastroenterology, Hepatology, and Nutrition (ESPGHAN) [10] and regarding the amounts of energy intakes that infants could have received per day [31]. Family pediatrician received further information by phone or videoconference if required. We summarized these recommendations in Table S1. In brief, we suggested to continue breastfeeding or formula along with the introduction of complementary food and to offer a variety of foods with different flavors and textures [10]. We recommended to introduce all variety of foods at the same time. We suggested to avoid delayed introduction of some foods to reduce possible allergy reactions. Amount of complementary food should be increased depending on child’s appetite. We recommended to start weaning from the 52 weeks of PMA and 9 months of corrected age only in the presence of the following of two criteria: (i) positive clinical judgment of family pediatrician to infants that attained motor skills (independently by growth and human milk alimentation) adequate to cope safely with solid foods and (ii) family deemed to be ready. In this way, we hypothesized a spontaneous distribution of the timing of weaning along the period between 52 weeks of PMA and 9 months of corrected age. During the follow-up visit, a researcher unaware of the study aims prospectively recorded time of complementary food introduction, and he kept in contact with the pediatrician to verify if parents respected the indication received regarding variety of foods, textures, frequency, and amounts of meals. The family pediatrician verified the compliance regarding recommendation received, including food texture, variety, frequency of meals, and caloric intake (Table S1). We reported data regarding compliance to our recommendations in a specific data form. We divided enrolled newborns in 2 groups according with the timing of weaning: (1) Early Weaning: introduction of complementary food before 6 months of corrected age and (2) Late Weaning: complementary food introduced after 6 months of corrected age. We excluded from analysis infants receiving complementary food before 52 weeks of PMA or after 9 months of corrected age.

Physician unaware of the study aims measured growth parameters (weight, length, BMI, and ponderal index) at 12 months of corrected age, and standardized growth parameters (weight z-score, length z-score, and BMI z-score) at 12 months were considered as primary outcome of the study.

### Statistics

Statistical analysis was performed using Statistical Package for Social Science software (SPSS Inc., Chicago, IL, USA) version 25.0. We checked for normality using Shapiro−Wilk test. The mean and 95% confidence interval summarized continuous variables and number and percentage described categorial variables. We used χ^2^ test for categorical variable and *t*-test or Mann−Whitney for paired and unpaired variables.

Correlations were performed between growth parameters at 12 months (standardized and unstandardized) and timing of weaning by Pearson correlation.

We performed linear regression analysis separately for male and female infants, using as covariates maternal age, gestational age at birth, duration of breastfeeding, age of weaning, and, as dependent variable, standardized growth parameters at 12 months. We also evaluated in a binary regression analysis the influence of covariates (intrauterine growth restriction, born before 28 weeks of PMA, male sex, morbidity, EUGR, exclusive breastfeeding up to weaning, and group assignment) on growth impairment at 12 months of corrected age (defined as Z-Score parameters < −1 for body weight, length and BMI).

We considered the level of significance for statistical tests as 2-sided (*p*-value < 0.05).

## 3. Results

**Figure 1:**
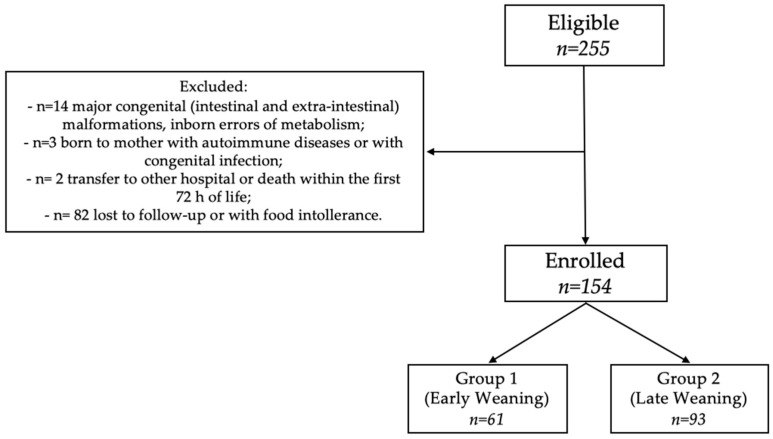
Flow-chart.

Of 255 eligible newborns, we enrolled 154 neonates at 12 months of corrected age (Figure 1). As shown in Table 1 and Table 2, the two groups of the study were similar for baseline clinical characteristics and morbidity rate.

Table 3 summarizes nutritional management of the newborns from birth. The rate of newborns receiving exclusive breast milk up to weaning were higher in group 1 compared to group 2 (Table 3). The mean age of weaning of total population was 6 months ± 1, and it appeared lower in group 1 (Table 3).

There were no differences for growth parameters (body weight, length, BMI, and ponderal index) at 52 weeks of PMA (Table S2). As shown in Table 4, the two groups did not show difference for growth parameters at 12 months of corrected age. To evaluate the influence of prematurity-related morbidity conditions on growth parameters, we separated all enrolled infants in two groups in relation to the presence of at least one prematurity-related morbidity conditions, and we found no statistical differences for growth parameters between the two subgroups at 52 weeks of PMA and 12 months of corrected age (Table S3).

We also observed no correlation between age of weaning and both unstandardized (body weight: r = 0.127, *p* = 0.118; length: r = 0.013, *p* = 0.873; BMI: r = 0.136, *p* = 0.091; ponderal index: r = 0.101, *p* = 0.212) and standardized (body weight: r = 0.153, *p* = 0.058; length: r = 0.056, *p* = 0.490; BMI: r = 0.143, *p* = 0.077) growth parameters at 12 months of corrected age.

Linear regression analysis, performed separately for male and female infants, showed that introduction of complementary food did not influence the standardized growth parameters at 12 months of corrected age (Table 5). Binary logistic regression model showed that early or late weaning did not influence growth parameters at 12 months of corrected age (Table 6).

Compliance of the recommendations provided at discharge on weaning was very high in the two study groups. In particular, all the children received complementary food between 52 weeks of PMA and nine months of corrected age. No baby received during weaning foods different from suggested in variety, texture, and amounts. All children enrolled in both groups received complementary food and caloric intakes as recommended.

## 4. Discussion

We demonstrated that the timing of weaning did not influence growth of VLBW infants at 12 months corrected age. These results were confirmed also after adjusting for confounding variables in linear and binary multivariate models.

For healthy, full-term newborns, several randomized control trials have reported no effects of the timing of weaning on long-term growth [32,33,34]. Observational studies have evaluated the relation between the timing of introduction of solid foods and obesity [35,36]. Grote et al. [37], in a prospective cohort study, demonstrated an increase in standardized weight and length and a worse BMI at 24 months of life in children born at term receiving semisolid foods early in life (between 14 and 17 weeks after birth). A systematic review [38] concluded that, in full-term neonates, the introduction of solids prior to four months of age may result in an increased risk of childhood obesity, but there is little evidence of adverse weight status outcomes associated with introducing solids at 4–6 rather than at six months.

There is no consensus for the weaning program of babies born preterm [10], and only few studies have evaluated growth parameters in preterm infants in relation with age of introduction of solid food. [39,40]. A randomized clinical trial (RCT) [39] including neonates born preterm with a mean gestational age of 31 weeks demonstrated that a preterm weaning strategy with early introduction of semisolid food soon after 13 weeks had a significant positive effect on length scores at 12 months compared to the control group, in which semisolid food was introduced after 17 weeks of postnatal age [39]. No significant difference was observed on weight and BMI of enrolled infants. The preterm weaning strategy also recommended the use of solid foods with a higher energy density and protein content compared to the control group. Both groups studied by Marriot et al. [39] started complementary feeding earlier compared to the weaning strategy adopted in our study. The Early Weaning group in the aforementioned study received complementary feeding earlier (12–13 weeks) compared to our study design. Patients in the two groups of this RCT, differently from our study, received complementary feeding with a different nutritional intake. Thus, it is not possible to establish if these results depended on nutritional intake or on timing of introduction of complementary feeding.

Morgan et al. [40] described the effects of the timing of weaning (before or after 12 weeks of life) on growth outcomes of infants born preterm up to 18 months of life. The authors showed that early weaned infants remained significantly heavier than later weaned infants evaluated at nine months [40]. However, this difference was not found at 18 months post-term follow-up.

We observed that the rate of infants receiving exclusively breast milk at the time when weaning was started was higher in group 1 compared to group 2. These results can be considered a casual association secondary to different time of solid food introduction. In other words, late introduction of solid food increases the probability of the not exclusive use of breast milk.

Our study suggested that weaning does not influence growth in the first year of life. In preterm infants, short- and long-term outcomes are influenced mainly by the nutritional approach of the first weeks of life [2,3,5,6,13,41], especially PN, while timing of weaning as a marginal impact on growth on this vulnerable population. In preterm newborns, a critical window for nutritional intervention remains in the first month of life. This could be due to the critical phase of the first weeks of life of these critical newborns, a window which appears to be more important and vulnerable compared to the timing of weaning. In fact, preterm infants are born at a time when their organs are structurally and functionally immature, leading to alterations in key organs as a result of the altered physical and biochemical environment caused by preterm birth [42,43,44].

Despite the interesting results regarding this yet undefined topic for preterm neonates, our findings should be interpreted considering some limitations. Results may be related to the effects of random error, bias, or confounding factors. We adjusted results for confounding variables that could have influenced the outcome of the study. However, confounding variables still unknown or not considered in our statistical model may have influenced the results. This was not an RCT. However, to reduce drop out, for randomization of the timing of weaning should be taken into account the two criteria that we used in this study. In our policy, the timing of weaning was guided by the presence of adequate infants motor skills and family collaboration. The researcher involved in the study did not participate in the choice of time of weaning. Consequently, we observed a spontaneous distribution of timing of weaning between 52 weeks of PMA and nine months. This methodology represents a selection bias that should be considered for the generalizability of the results. We verified that the infants received the caloric intakes recommended for age [31]. All enrolled infants respected the recommendations regarding the energy intakes. No difference in compliance regarding this aspect was found between the two study groups. Thus, we hypothesize that energy intake was similar between the two study groups; however, the lack of information about individual energy intakes represent an important limitation of the study. Further trials are advocated to evaluate if energy intakes, derived from milk and complementary food, may have a significant impact on growth in infants born preterm. We adjusted results in multivariate analyses for neonatal variables that could have influenced the outcome. However, it is not feasible to select all the possible variables that could influence growth of children in the first year of life; thus, we focused on variables relating to the neonatal period that are the most important for long-term consequences in preterm infants. Prematurity-related morbidity conditions could influence growth parameters; however, in univariate and multivariate models, we found no association between morbidity and growth at 12 months corrected age. Also, growth parameters at the beginning of weaning could influence the results at 12 months. We did not analyze data on growth at the beginning of weaning for each patient but at 52 weeks of PMA for all enrolled children. Despite that this could have an influence on our primary outcome, data recorded at 52 weeks of PMA did not show differences between the two study groups. To limit selection bias, neonatologists evaluating eligibility used objective inclusion criteria (such as gestational age and birth weight), unaware of the study aims, and data for the statistical analysis were collected by researchers not involved in the eligibility assessment and clinical practice, unaware of the study outcomes and design. We discussed and defined a protocol for the collection, measurement, and interpretation of data before starting the study. Besides, a blinded statistician performed the data analysis. Additional limitation of the study is the lack of information regarding strategy, quality, and quantity of complementary feeding and follow-up limited at 12 months of life.

## 5. Conclusions

In conclusion, our data demonstrated that the timing of weaning did not influence growth in the first year of life. The strategy of weaning, including the quality and quantity of semisolid food introduction, together with the timing might influence long-term growth outcomes. Thus, further well designed RCTs establishing the optimal timing and strategy of weaning for infants born preterm are advocated.

## Figures and Tables

**Table 1:** Baseline clinical characteristics of the study population.

	Group 1 (Early Weaning) (*n* *=* 61)	Group 2 (Late Weaning) (*n* *=* 93)
Maternal age, years	36 (34 to 37)	35 (35 to 36)
Intrauterine growth restriction, No (%)	5 (8.2)	11 (11.8)
Birth weight, grams	1337 (1248 to 1427)	1249 (1184 to 1313)
Gestational age, week	29 (29 to 30)	29 (29 to 30)
Small for gestational age at birth, No (%)	10 (16.4)	17 (18.3)
Antenatal steroids ^a^, No. (%)	36 (59.0)	68 (73.1)
Cesarean section, No. (%)	53 (86.9)	85 (91.4)
Male sex, No. (%)	36 (59.0)	43 (46.2)
Twins, No. (%)	20 (32.8)	35 (37.6)
5-min Apgar score	8 (7 to 8)	8 (7 to 8)
pH at birth	7.3 (7.2 to 7.3)	7.3 (7.2 to 7.3)
CRIB II score	5 (5 to 6)	6 (5 to 7)
Extrauterine growth restriction at 36 weeks of PMA, No. (%)	43 (70.5)	66 (71.0)

Table legend. ^a^ Intramuscular steroid cycle in two doses of 12 mg over a 24-h period; CRIB (clinical risk index for babies); PMA (postmenstrual age). Data are shown as mean (95% confidence interval) when not specified.

**Table 2:** Morbidity rate of the study population.

	Group 1 (Early Weaning) (*n* *=* 61)	Group 2 (Late Weaning) (*n* *=* 93)
Morbidity ^a^	15 (24.6)	26 (28.0)
Necrotizing enterocolitis	2 (3.3)	3 (3.2)
Intraventricular hemorrhage	1 (1.6)	4 (4.3)
Periventricular Leukomalacia	0 (0.0)	2 (2.2)
Sepsis proven by positive cultures	1 (1.6)	6 (6.5)
Retinopathy of prematurity	11 (18.0)	18 (19.4)
Bronchopulmonary dysplasia	3 (4.9)	3 (3.2)
Invasive mechanical ventilation	20 (32.8)	21 (22.6)
Non-invasive mechanical ventilation	45 (73.8)	74 (79.8)

Table legend. ^a^ at least one prematurity-related morbidity (necrotizing enterocolitis or intraventricular hemorrhage or periventricular leukomalacia or sepsis proven by positive cultures or retinopathy of prematurity or bronchopulmonary dysplasia). Data are shown as number (percentage).

**Table 3:** Nutritional management of the study population.

	Group 1 (Early Weaning) (*n* *=* 61)	Group 2 (Late Weaning) (*n* *=* 93)
Full enteral feeding, days of life	14 (11 to 17)	14 (11 to 18)
Start of enteral nutrition before 72 h of life, No. (%)	50 (82.0)	81 (87.1)
Duration of parenteral nutrition, days	13 (10 to 15)	12 (10 to 14)
Exclusive breastfeeding up to weaning, No. (%)	5 (8.2) *	1 (1.1)
Exclusive infant formula up to weaning, No. (%)	14 (23.0)	15 (16.1)
Duration of breastfeeding, weeks	4 (2 to 6)	5 (2 to 8)
Age of weaning, months	4 (4 to 5) *	6 (6 to 7)

Notes. * *p*-value < 0.05 vs. group 2. Data are shown as mean (95% confidence interval) when not specified.

**Table 4:** Growth parameters at 12 months of corrected age.

	Group 1 (Early Weaning) (*n* = 61)	Group 2 (Late Weaning) (*n* = 93)
Body weight, grams	8848 (8444 to 9251)	9031 (8746 to 9317)
Body weight Z-Score	−0.6 (−1.0 to −0.1)	−0.3 (−0.5 to −0.1)
Length, cm	74.3 (73.4 to 75.1)	73.5 (72.6 to 74.4)
Length Z-Score	0 (−0,4 to 0.3)	−0.2 (−0.5 to 0.1)
Body mass index	16 (15 to 17)	17 (16 to 17)
Body mass index Z-Score	−0.8 (−1.3 to −0.2)	−0.2 (−0.5 to 0)
Ponderal Index	22 (21 to 23)	23 (22 to 23)

Notes. Data are shown as mean (95% confidence interval).

**Table 5:** Linear regression analysis to evaluate the influence of confounding variables on primary outcome.

	Dependent Variables	Confounding Variables	B	*p*-Value	β	95% Confidence Interval
Male newborns	Body weight Z-Score	Maternal age	−0.023	0.542	−0.613	−0.097 to 0.051
		Gestational age at birth	−0.013	0.906	−0.119	−0.235 to 0.209
		Duration of breastfeeding	0.000	0.995	0.007	−0.064 to 0.065
		Age of weaning	0.256	0.063	1.890	−0.014 to 0.526
	Length Z-Score	Maternal age	0.018	0.530	0.631	−0.040 to 0.077
		Gestational age at birth	0.012	0.894	0.133	−0.162 to 0.186
		Duration of breastfeeding	−0.019	0.449	−0.762	−0.070 to 0.031
		Age of weaning	0.098	0.358	0.926	−0.113 to 0.310
	Body mass index Z-Score	Maternal age	−0.043	0.362	−0.917	−0.136 to 0.050
		Gestational age at birth	−0.026	0.850	−0.190	−0.304 to 0.251
		Duration of breastfeeding	0.013	0.741	0.331	−0.067 to 0.094
		Age of weaning	0.254	0.137	1.503	−0.083 to 0.251
Female newborns	Body weight Z-Score	Maternal age	0.008	0.775	0.288	−0.049 to 0.066
		Gestational age at birth	−0.004	0.947	−0.067	−0.133 to 0.124
		Duration of breastfeeding	0.006	0.586	0.547	−0.015 to 0.026
		Age of weaning	0.015	0.832	0.213	−0.123 to 0.152
	Length Z-Score	Maternal age	0.036	0.285	1.078	−0.031 to 0.103
		Gestational age at birth	−0.053	0.484	−0.704	−0.203 to 0.097
		Duration of breastfeeding	0.007	0.540	0.616	−0.106 to 0.031
		Age of weaning	−0.010	0.901	−0.124	−0.171 to 0.151
	Body mass index Z-Score	Maternal age	−0.019	0.482	−0.708	−0.073 to 0.035
		Gestational age at birth	0.034	0.569	0.573	−0.085 to 0.154
		Duration of breastfeeding	0.001	0.897	0.131	−0.018 to 0.020
		Age of weaning	0.034	0.600	0.527	−0.094 to 0.162

**Table 6:** Binary logistic regression analysis to evaluate the influence of confounding variables on primary outcome.

Dependent Variables (Not or Yes)	Confounding Variables (Not or Yes)	B	*p*-Value	O.R. (95% Confidence Interval)
Body weightZ-Score < −1	Intrauterine growth restriction	0.338	0.574	1.402 (0.432 to 4.549)
	Born before 28 weeks of gestational age	0.354	0.407	1.425 (0.617 to 3.292)
	Male sex	0.003	0.993	1.003 (0.468 to 2.149)
	Morbidity ^a^	0.169	0.695	1.184 (0.509 to 2.754)
	Extrauterine growth restriction	0.604	0.186	1.830 (0.747 to 4.483)
	Exclusive breastfeeding up to weaning	0.127	0.890	1.135 (0.188 to 6.836)
	Group 2 (Late weaning)	−0.374	0.332	0.688 (0.323 to 1.464)
LengthZ-Score < −1	Intrauterine growth restriction	0.739	0.202	2.094 (0.673 to 6.515)
	Born before 28 weeks of gestational age	0.273	0.545	1.314 (0.542 to 3.185)
	Male sex	0.246	0.541	1.280 (0.581 to 2.819)
	Morbidity ^a^	−0.320	0.496	0.726 (0.289 to 1.825)
	Extrauterine growth restriction	0.561	0.228	1.753 (0.704 to 4.364)
	Exclusive breastfeeding up to weaning	0.181	0.844	1.198 (0.197 to 7.294)
	Group 2 (Late weaning)	−0.304	0.446	0.738 (0.338 to 1.613)
Body mass indexZ-Score < −1	Intrauterine growth restriction	−0.527	0.447	0.591 (0.152 to 2.293)
	Born before 28 weeks of gestational age	−0.173	0.702	0.841 (0.347 to 2.040)
	Male sex	0.365	0.353	1.440 (0.667 to 3.111)
	Morbidity ^a^	0.347	0.428	1.414 (0.600 to 3.334)
	Extrauterine growth restriction	0.667	0.151	1.948 (0.783 to 4.846)
	Exclusive breastfeeding up to weaning	−0.974	0.397	0.378 (0.040 to 3.598)
	Group 2 (Late weaning)	−0.584	0.131	0.558 (0.261 to 1.191)

Notes. ^a^ at least one prematurity-related morbidity (necrotizing enterocolitis or intraventricular hemorrhage or periventricular leukomalacia or sepsis proven by positive cultures or retinopathy of prematurity or bronchopulmonary dysplasia).

## Data Availability

Data are available upon reasonable request. All data relevant to the study are included in the article. Access to raw data would be provided upon request.

## Supplementary Materials

The following are available online at www.mdpi.com/article/10.3390/children8121085/s1, Table S1. Practical guidance for weaning between 3 to 12 months of corrected age. Table S2. Growth parameters at 52 weeks of postmenstrual age. Table S3. Growth parameters in relation to the presence of morbidity conditions.
