# Supplementary material for: Complementary Feeding and Growth in Infants Born Preterm: A 12 Months Follow-Up Study
Source: Children (Basel). 2021 Nov 24;8(12):1085. doi: 10.3390/children8121085 (PMC8700469; doi:10.3390/children8121085)
Supplement: Supplementary file 1 [file children-08-01085-s001.zip › children-1344263-supplementary.pdf]

Table S1. Practical guidance for weaning between 3 to 12 months of corrected age.

	Variety and Texture	Frequency
**3-9 months of corrected age**	Continue breastfeeding<br><br>Start with pudding made out of cereal flour (rice, wheat, corn, semolina) or roots (cassava, potatoes) mixed with vegetable broth, add extra virgin olive oil<br><br>Mashed family foods (made out of cereal flour or roots mixed with fish or meat, pulses, vegetables, eggs, cheese); add extra virgin olive oil	2–3 meals per day depending on the child's appetite;<br><br>1–2 nutritious snacks may be offered: available fruits, yogurt, milk, bread
**9-12 months of corrected age**	Continue breastfeeding;<br><br>Mashed family foods (made out of cereal flour or roots mixed with fish or meat, pulses, vegetables, eggs, cheese); add extra virgin olive oil<br><br>Chopped foods that babies can pick up and feed themselves	3–4 meals per day depending on the child's appetite;<br><br>1–2 nutritious snacks may be offered: available fruits, yogurt, milk, bread

Table S2. Growth parameters at 52 weeks of postmenstrual age.

	Group 1 (Early Weaning) (n. 61)	Group 2 (Late Weaning) (n.93)
Body weight, grams	5880 (5618 to 6142)	5715 (5492 to 5939)
Body weight Z-Score	−0.4 (−0.8 to −0.1)	−0.4 (−0.7 to −0.1)
Length, cm	59.8 (58.7 to 61.0)	58.5 (57.6 to 61.0)
Length Z-Score	−0.4 (−1.0 to 0.1)	−0.7 (−1.1 to −0.3)
Body mass index	16 (16 to 17)	17 (16 to 17)
Body mass index Z-Score	−0.2 (−0.6 to 0.1)	0.1 (−0.3 to 0.5)
Ponderal Index	28 (26 to 29)	29 (27 to 31)

Notes. Data were showed as mean (95% confidence interval).

Table S3 Growth parameters in relation to the presence of morbidity conditions.

	With morbidity conditions (n. 41)	Without morbidity conditions (n.113)
52 weeks of postmenstrual age		
Body weight, grams	5728 (5422 to 6034)	5799 (5593 to 6004)
Body weight Z-Score	−0.4 (−0.8 to 0)	−0.4 (−0.7 to −0.1)
Length, cm	58.8 (57.5 to 60.0)	59.1 (58.2 to 60.0)
Length Z-Score	−0.6 to (−1.2 to −0.1)	−0.6 (−1.0 to −0.2)
Body mass index	17 (16 to 17)	17 (16 to 17)
Body mass index Z-Score	0 (−0.5 to 0.4)	0 (−0.4 to 0.3)
Ponderal Index	28 (27 to 30)	28 (27 to 30)
12 months of corrected age		
Body weight, grams	8830 (8435 to 9225)	9005 (8719 to 9291)
Body weight Z-Score	−0.6 (−0.9 to −0.2)	−0.4 (−0.6 to −0.1)
Length, cm	73.8 (72.4 to 75.2)	73.8 (73.0 to 74.5)
Length Z-Score	−0.2 (−0.6 to 0.3)	−0.1 (−0.4 to 0.1)
Body mass index	16 (16 to 17)	17 (16 to 17)
Body mass index Z-Score	−0.6 (−1.0 to −0.2)	−0.4 (−0.7 to 0)
Ponderal Index	22 (21 to 23)	22 (21 to 23)

Notes. Data were showed as mean (95 % confidence interval).
